# Foot related impairments and disability in juvenile idiopathic arthritis persist despite modern day treatment paradigms

**DOI:** 10.1186/1757-1146-4-S1-P24

**Published:** 2011-05-20

**Authors:** Gordon J Hendry, Janet Gardner-Medwin, Gordon F Watt, Jim Woodburn, John H McColl, Roger D Sturrock

**Affiliations:** 1 School of Biomedical and Health Sciences, University of Western Sydney, Sydney, Locked Bag 1797, Australia; 2 Glasgow Caledonian University, Glasgow, G4 0BA, UK; 3 University of Glasgow, Glasgow, G12 8QQ, UK

## Background

Foot problems such as synovitis, growth disturbance and deformity are considered common in juvenile idiopathic arthritis (JIA) and have been previously reported in over 90% of cases. The medical management of JIA appears to have improved recently however little is known about the impact of new regimes on localised joints such as in the foot. This pilot study aimed to investigate the prevalence of foot related impairments and disability, and survey the medical and podiatric management of patients in a cohort of UK children with JIA.

## Methods

This study was a tertiary care based cross-sectional survey. Thirty consecutive JIA patients with a history of foot and ankle arthritis completed the juvenile arthritis foot disability index questionnaire (JAFI) (0-4 for each domain), child health assessment questionnaire (CHAQ) (0-3), and pain visual analogue scale (VAS) (0-100mm). Foot deformity score (0-38), active and limited joint counts (0-77) and walking speed (m/s) were measured also recorded. Foot care provision over the previous 12 months was determined from the medical records in 23/30 participants. Results were analysed using simple descriptive statistics and expressed as median (range).

## Results

Children received biologic agents in 35%, DMARDs in 65%, and 90% of participants had received multiple intra-articular cortico-steroid injections. Median (range) values for foot disease outcomes were JAFI impairment = 1 (0-3), JAFI activity limitation = 1 (0-4) JAFI participation restriction 1 (0-3), CHAQ = 0.38 (0-2), VAS pain = 22 (0-79), foot deformity = 6 (0-20), active joints = 0 (0-7), limited joints (0-31), walking speed = 1.09 (0.84-1.38). The JAFI scores represent mild foot related impairment and disability. Gait defects, deformity or abnormal foot posture, and/or active foot disease were the main reasons for referral. 43% of children received specialist podiatry care comprising footwear advice, orthotic therapy, and silicone digital appliances together with intrinsic muscle strengthening exercises.

## Conclusions

Despite DMARD/biologic regimes and specialist podiatry, foot related impairment and disability persists in JIA children. Foot care appears to be in line with current recommendations. Further study is required to determine the long-term consequences of these changes found during childhood in the foot.

